# The impact of obesity on left ventricular hypertrophy and diastolic dysfunction in children and adolescents

**DOI:** 10.1038/s41598-021-92463-x

**Published:** 2021-06-22

**Authors:** Joanna Bartkowiak, Ernest Spitzer, Reto Kurmann, Fabian Zürcher, Peter Krähenmann, Victoria Garcia-Ruiz, Jorge Mercado, Christoph Ryffel, Sylvain Losdat, Nassip Llerena, Pedro Torres, Jonas Lanz, Martin Stocker, Ben Ren, Martin Glöckler, Thomas Pilgrim

**Affiliations:** 1 Department of Cardiology, Inselspital, Bern University Hospital, University of Bern, 3010 Bern, Switzerland; 2 Cardialysis, Rotterdam, The Netherlands; 3 Thoraxcenter, Erasmus University Medical Center, Rotterdam, The Netherlands; 4 Department of Cardiology, Cantonal Hospital Lucerne, Lucerne, Switzerland; 5 Department of Cardiology, Cantonal Hospital St. Gallen, St. Gallen, Switzerland; 6 Institute of Cardiology CardioSalud, Arequipa, Peru; 7 CTU Bern, University of Bern, Bern, Switzerland; 8 National Hospital Carlos Alberto Seguín Escobedo, Arequipa, Peru; 9 Department of Pediatric Cardiology, Inselspital, University of Bern, Bern, Switzerland

**Keywords:** Cardiology, Risk factors

## Abstract

Childhood obesity continues to escalate worldwide and may affect left ventricular (LV) geometry and function. The aim of this study was to investigate the impact of obesity on prevalence of left ventricular hypertrophy (LVH) and diastolic dysfunction in children. In this analysis of prospectively collected cross-sectional data of children between 5 and 16 years of age from randomly selected schools in Peru, parameters of LV geometry and function were compared according to presence or absence of obesity (body mass index z-score > 2). LVH was based on left ventricular mass index (LVMI) adjusted for age and sex and defined by a z-score of > 2. LV diastolic function was assessed using mitral inflow early-to-late diastolic flow (E/A) ratio, peak early diastolic tissue velocities of the lateral mitral annulus (E′), early diastolic transmitral flow velocity to tissue Doppler mitral annular early diastolic velocity (E/E′) ratio, and left atrial volume index (LAVI). Among 1023 children, 681 children (mean age 12.2 ± 3.1 years, 341 male (50.1%)) were available for the present analysis, of which 150 (22.0%) were obese. LVH was found in 21 (14.0%) obese and in 19 (3.6%) non-obese children (p_adjusted_ < 0.001). LVMI was greater in obese than that in non-obese children (36.1 ± 8.6 versus 28.7 ± 6.9 g/m^2.7^, p < 0.001). The mean mitral E/E′ ratio and LAVI were significantly higher in obese than those in non-obese individuals (E/E′: 5.2 ± 1.1 versus 4.9 ± 0.8, p_adjusted_ = 0.043; LAVI 11.0 ± 3.2 versus 9.6 ± 2.9, p_adjusted_ = 0.001), whereas E′ and E/A ratio were comparable. Childhood obesity was associated with left ventricular hypertrophy and determinants of diastolic dysfunction.

ClinicalTrials.gov Identifier: NCT02353663.

## Introduction

Childhood obesity has been emerging as a global epidemic in recent decades^1^. Worldwide, more than 340 million children and adolescents aged 5–16 years are deemed overweight or obese^2^. Data from a prospective cohort study in Sweden indicated that the mortality rate of children with obesity is three times higher than a population-based comparison group once they reach early adulthood^3^.

Accumulating evidence suggests that obesity in early childhood may significantly affect cardiac geometry and function^4^. Several small studies indicated differences in left ventricular (LV) mass, left atrial size and LV diastolic function between obese and non-obese children^5–7^. In adults, hemodynamic and metabolic changes associated with obesity have been shown to promote the development of left ventricular hypertrophy (LVH) and impaired LV diastolic function^8,9^, eventually leading to heart failure with preserved ejection fraction (HFpEF)^10^. Prevalence of HFpEF exceeds prevalence of heart failure with reduced ejection fraction, is less amenable to medical treatment, and carries a high risk of adverse outcome with a mortality rate exceeding 50% 5 years after the first episode^11,12^. Prevention of LVH and diastolic dysfunction takes therefore center stage to mitigate progression to HFpEF.

Available evidence on a potential correlation between childhood obesity and prevalence of LVH and diastolic dysfunction is scarce. The aim of the present study was to test the hypothesis that children with obesity are at increased risk of LVH and diastolic dysfunction as compared with normal-weight children.

## Methods

### Study population

We analyzed prospectively collected clinical and echocardiographic data of children 5–16 years of age from randomly selected schools in Arequipa, Peru. The sampling frame consisted of 457 primary and secondary schools. Forty classes from 20 schools were randomly selected using multistage sampling and taking into account location (urban versus rural) and administration (public or private) of the schools. All children attending one of the selected classes were eligible for inclusion. For the purpose of the present analysis, children with structural heart defects or more than mild valvular lesions, missing height or weight or missing echocardiographic parameters required for the assessment of LVH or diastolic function were excluded. The cross-sectional study was approved by the Human Research Ethics Committee of the University San Martín de Porres, Lima, Peru (Officio No. 48-2014-CIEI-USMP-CCM), and local authorizations were granted by the Regional Administrations of the Health and Education Ministries. Written informed consent for participation in the survey was obtained from parents or guardians, and children. All methods were performed in accordance with the relevant guidelines and regulations.

### Data collection

Details of data acquisition and echocardiographic evaluation have been reported previously^13^. In brief, demographic characteristics were collected using standardized interviews customized to the age of the children, and were followed by a focused physical examination. Transthoracic echocardiography was performed by a trained cardiologist with a MyLabAlpha (Esaote, Italy) portable echocardiography machine using a systematic acquisition protocol detailed previously^13^. Recorded echocardiographic raw data was analyzed in the academic imaging core lab at Bern University Hospital by four imaging experts (ES, RK, FZ, PK) and in the academic imaging core lab at Cardialysis (ES, VG) in Rotterdam. All data was entered into a dedicated database maintained at the Clinical Trials Unit of the University of Bern, Switzerland.

### Definitions

Obesity was defined according to World Health Organization (WHO) criteria for individuals < 19 years of age based on z-score of the body mass index (BMI). Calculation of the z-score for BMI is specified in the online—only “Supplemental material S1”. Obesity was defined by z-score > 2 of the BMI.

Left ventricular mass (LVM) was calculated using LV wall thickness and LV cavity size measured during diastole. LVM was then indexed to height, as recommended by the National High Blood Pressure Education Program Working Group on High Blood Pressure in Children and Adolescents^14^. The calculation of LVM and left ventricular mass index (LVMI) is specified in the online—only “Supplemental material S1”. LVH was defined as any LVMI value above the 95th percentile. In children > 9 years of age, values > 40 g/m^2.7^ in girls and > 45 g/m^2.7^ in boys were considered abnormal (i.e., > 95th percentile) because of little variation. In children ≤ 9 years of age, LVH was recorded if LVMI was > 95 percentile based on published reference^15^. Left atrial volume was obtained in the apical four- and two-chamber views and indexed to the 2.7 power of height in meter. LV diastolic function was assessed by pulse-wave and tissue Doppler in the apical four-chamber view. Mitral inflow early-to-late diastolic flow (E/A) ratio, peak early diastolic tissue velocity at the mitral lateral annulus (E′) and early diastolic transmitral flow velocity to tissue Doppler mitral annular early diastolic velocity (mitral E/E′) ratio were recorded. Left ventricular end-diastolic volume (LVEDV) and left ventricular end-systolic volume (LVESV) were calculated using the Simpson’s biplane method of disks summation technique according to the American Society of Echocardiography recommendations^16^. LV systolic function was expressed as ejection fraction (EF), derived from the LVEDV and LVESV. Additionally, tissue Doppler-derived peak systolic velocity of LV wall (LV S′) measured at the mitral lateral annulus was recorded. Right ventricular function was assessed with tricuspid annular plane systolic excursion (TAPSE) and tissue Doppler-derived peak systolic velocity at RV free wall (RV S′).

### Statistical analysis

Continuous variables are presented as mean (standard deviation) or median (lower quartile, upper quartile) depending on the distribution and categorical variables as counts (%). Obese and non-obese groups were compared using Chi-square test, Fisher’s exact test, Student’s t-test or Wilcoxon–Mann–Whitney test, as appropriate. For variables related to diastolic function, we additionally reported p-values adjusted for child age and child sex, computed using linear regression models that included age and sex as covariates. We also performed a propensity score matched analysis based on age and sex using ≥ 5 neighbours. Significance tests were two-tailed with a significance level set to 0.05. All analyses were conducted in Stata 16 (StataCorp. 2019. Stata Statistical Software: Release 16. College Station, TX: StataCorp LLC).

## Results

### Study population

**Figure 1:**
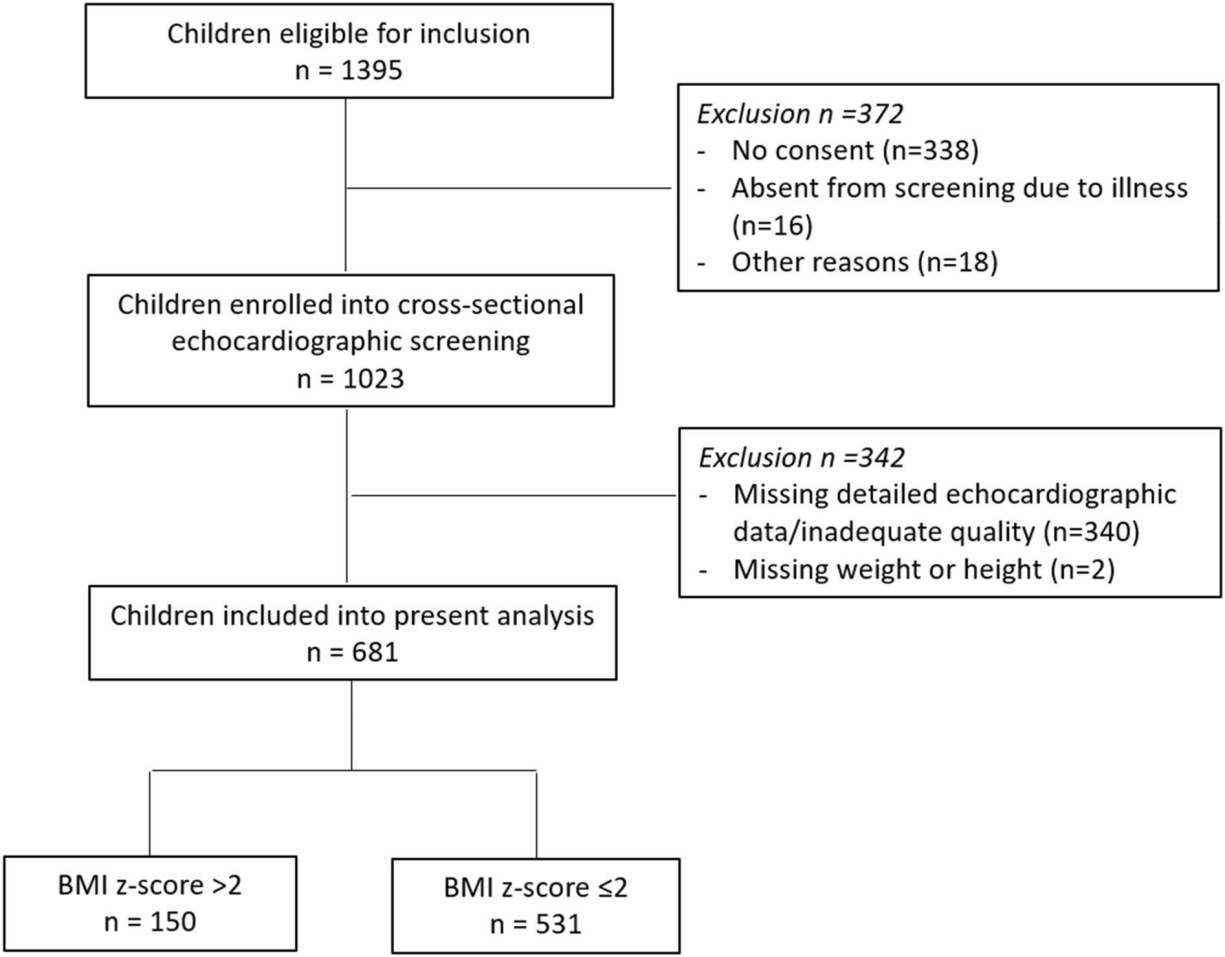
Study flow chart. The study flow chart illustrates the number of eligible children and the number of children included for the purpose of the present analysis.

Among 1023 children enrolled between April and May 2014, 342 subjects were excluded because of structural heart disease, incomplete echocardiographic data or missing BMI values, and 681 individuals (mean age 12.2 ± 3.1 years, 341 male (50.1%)) remained for the purpose of the present analysis (Fig. 1). Baseline characteristics of obese (n = 150, 22.0%) and non-obese children (n = 531, 78.0%) are summarized in Table 1. Obese children were younger than non-obese children (10.6 ± 2.4 years versus 12.6 ± 3.1 years, p < 0.001) and more commonly male (107 (71.3%) versus 234 (44.1%), p < 0.001).

**Table 1:** Baseline characteristic.

	All patients (n = 681)	Non-obese (BMIz ≤ 2) (n = 531)	Obese (BMIz > 2) (n = 150)	p-value
Age (months), mean (SD)	146 (36.7)	151 (36.9)	127 (29.0)	< 0.001
Sex (male), n (%)	341 (50.1%)	234 (44.1%)	107 (71.3%)	< 0.001
Height (cm), mean (SD)	142 (14.8)	144 (14.9)	138 (13.3)	< 0.00
Waist circumference (cm), mean (SD)	72.3 (9.38)	70.3 (8.12)	79.4 (10.1)	< 0.001
Heart rate (bpm), mean (SD)	79.4 (7.77)	79.1 (7.77)	80.2 (7.73)	0.132
Oxygen saturation (%), median (IQR)	96.0 [95.0–97.0]	96.0 [95.0–97.0]	96.0 [94.0–97.0]	0.061

*BMIz* BMI Z-score, *SD* standard deviation, *IQR* interquartile range.

### Cardiac geometry and function

**Figure 2:**
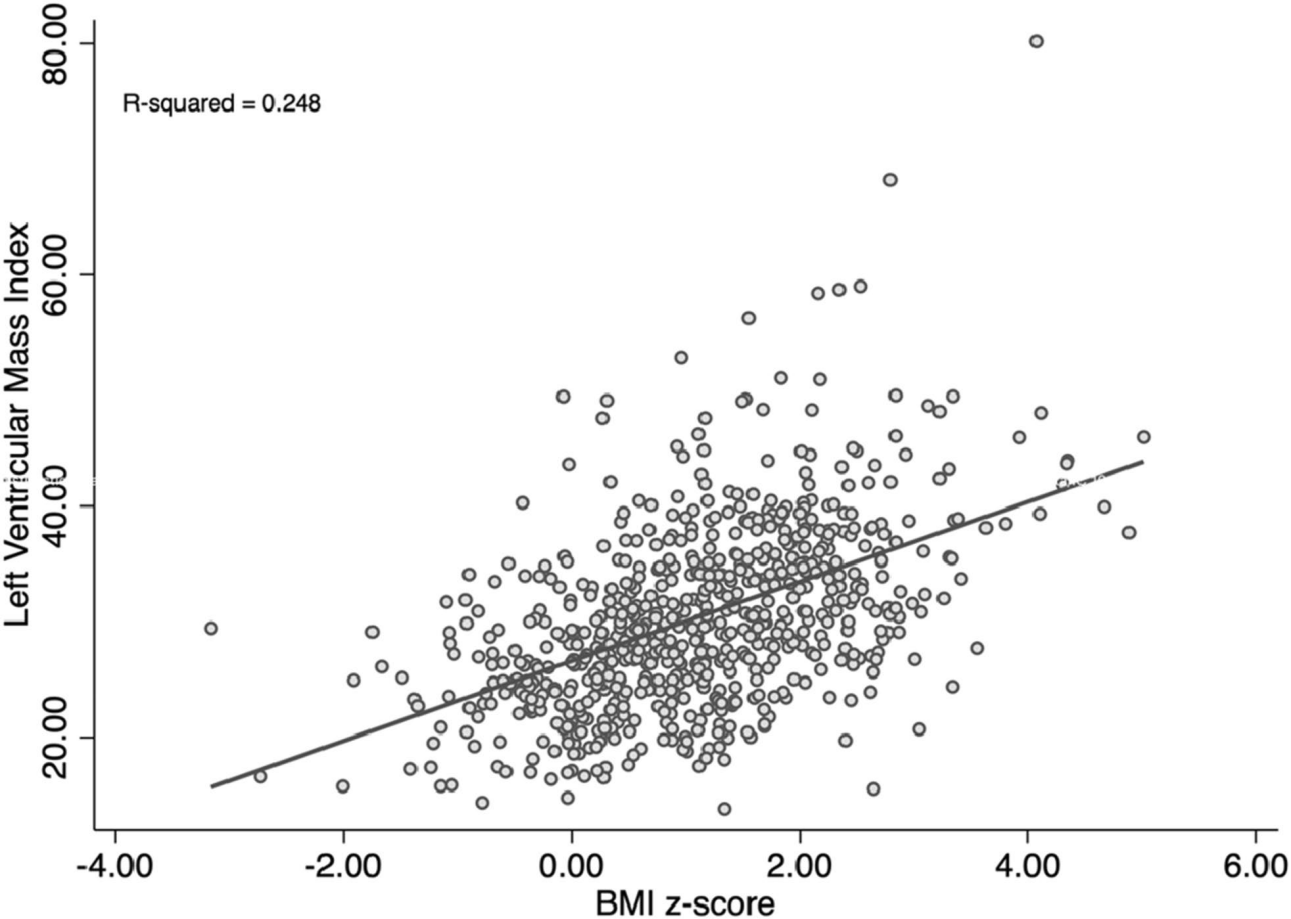
Relationship between left ventricular mass index and BMI z-score. The z-score of the body mass index (BMI) is shown on the x-axis. Left ventricular mass index (g/m^2.7^) is shown on the y-axis. Each grey dot represents one child. Grey line is the linear regression line.

**Figure 3:**
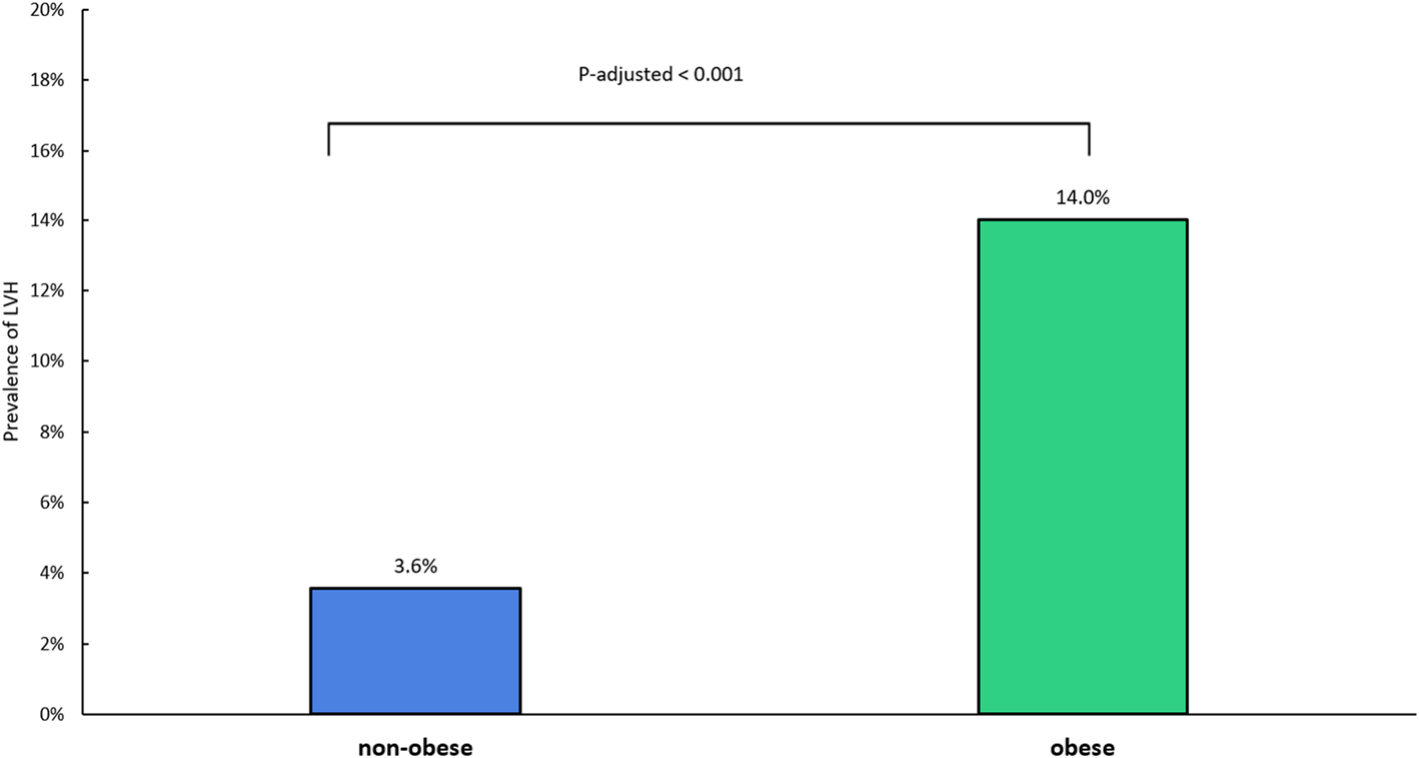
Bar graph illustrating prevalence of left ventricular hypertrophy (LVH) in obese and non-obese children.

Echocardiographic parameters of cardiac geometry and function are summarized in Table 2 Children with obesity were found to have greater relative wall thickness than non-obese children (0.352 ± 0.069 versus 0.332 ± 0.080, p = 0.005) as well as greater LVMI (36.1 ± 8.64 g/m^2.7^ versus 28.7 ± 6.93 g/m^2.7^, p < 0.001). There was a linear correlation between LVMI and BMI z-score (Fig. 2). Left ventricular hypertrophy was found in 21 (14.0%) obese and in 19 (3.6%) non-obese children (p_adjusted_ < 0.001) (Table 2, Fig. 3).

**Table 2:** Echocardiographic findings.

	All children (n = 681)	Non-obese (BMIz ≤ 2) (n = 531)	Obese (BMIz > 2) (n = 150)	p-value	p-value adj	p-value (multiple imputation) (n = 905)
**Cardiac geometry**						
LV Hypertrophy, n (%)	40 (5.87%)	19 (3.58%)	21 (14.0%)	< 0.001	< 0.001	< 0.001
LVEDD (cm), mean (SD)	3.91 (0.479)	3.91 (0.492)	3.94 (0.431)	0.479		
LVESD (cm), mean (SD)	2.39 (0.323)	2.40 (0.330)	2.38 (0.301)	0.576		
SWTd (cm), mean (SD)	0.731 (0.157)	0.718 (0.158)	0.778 (0.144)	< 0.001		
PWTd (cm), mean (SD)	0.648 (0.123)	0.638 (0.123)	0.686 (0.116)	< 0.001		
RWT, mean (SD)	0.336 (0.078)	0.332 (0.080)	0.352 (0.069)	0.005		
LVMI (g/m^2.7^), mean (SD)	30.4 (7.95)	28.7 (6.93)	36.1 (8.64)	< 0.001		
Right atrial area ES (cm^2^), mean (SD)	10.2 (2.32)	10.1 (2.29)	10.6 (2.37)	0.021		
Basal RV diameter 4C (cm), mean (SD)	3.07 (0.599)	3.05 (0.608)	3.14 (0.563)	0.151		
Base-to-apex lenght 4C (cm), mean (SD)	6.22 (0.947)	6.18 (0.936)	6.36 (0.975)	0.052		
Mid RV diameter 4C (cm), mean (SD)	2.78 (0.586)	2.76 (0.588)	2.86 (0.574)	0.196		
**Cardiac function**						
Systolic LV function						
LV S′ (cm/s), mean (SD)	12.1 (1.88)	12.2 (1.88)	12.0 (1.87)	0.202		
EF (%), mean (SD)	69.4 (6.29)	69.2 (6.28)	70.0 (6.30)	0.198		
Diastolic LV function						
E/E′ ratio, mean (SD)	4.99 (0.898)	4.93 (0.845)	5.18 (1.05)	0.003	0.043	0.073
E/E′ ≥ mean + 2 SD, n (%)	17 (2.50%)	10 (1.88%)	7 (4.67%)	0.072	0.120	
LAVI (ml/m^2.7^), mean (SD)	9.9 (3.03)	9.6 (2.91)	11.0 (3.17)	< 0.001	0.001	< 0.001
LAVI ≥ mean + 2 SD, n (%)	17 (2.50%)	8 (1.51%)	9 (6.0%)	0.006	0.038	
E′ (cm/s), mean (SD)	20.4 (3.08)	20.5 (2.96)	20.2 (3.46)	0.347	0.277	0.8998
E/A ratio, mean (SD)	1.77 (0.453)	1.78 (0.453)	1.77 (0.455)	0.855	0.271	0.651
RV function						
RV S′ (cm/s), mean (SD)	14.3 (1.83)	14.3 (1.86)	14.1 (1.70)	0.141		
TAPSE (cm), mean (SD)	2.12 (0.298)	2.13 (0.301)	2.09 (0.287)	0.096		
**Valvular heart disease**^**a**^						
Mild mitral regurgitation, n (%)	10 (1.47%)	8 (1.51%)	2 (1.33%)	1.000		
Mild tricuspid regurgitation, n (%)	93 (13.7%)	73 (13.7%)	20 (13.3%)	1.000		

*Adj. p value* p value adjusted for age and sex, *4C* four-chamber view, *BMIz* BMI z-score, *E* inflow early diastolic flow velocity, *E′* mitral lateral peak early diastolic tissue velocities, *EF* ejection fraction, *ES* end systolic, *E/A* inflow early-to-late diastolic flow ratio, *LAVI* left atrial volume index, *LV* left ventricle, *LVEDD* left ventricular end-diastolic diameter, *LVESD* left ventricular end-systolic diameter, *LVMI* left ventricular mass index, *LV S*′ tissue Doppler-derived peak systolic velocity of LV wall, *PWTd* posterior wall thickness (end diastolic), *RV* right ventricle, *RV S*′ tissue Doppler-derived peak systolic velocity at RV free wall, *RWT* relative wall thickness, *SWTd* septal wall thickness (end diastolic), *sd* standard deviation, *TAPSE* tricuspid annular plane systolic excursion.

^a^Patients with > mild valvular lesions were excluded.

**Figure 4:**
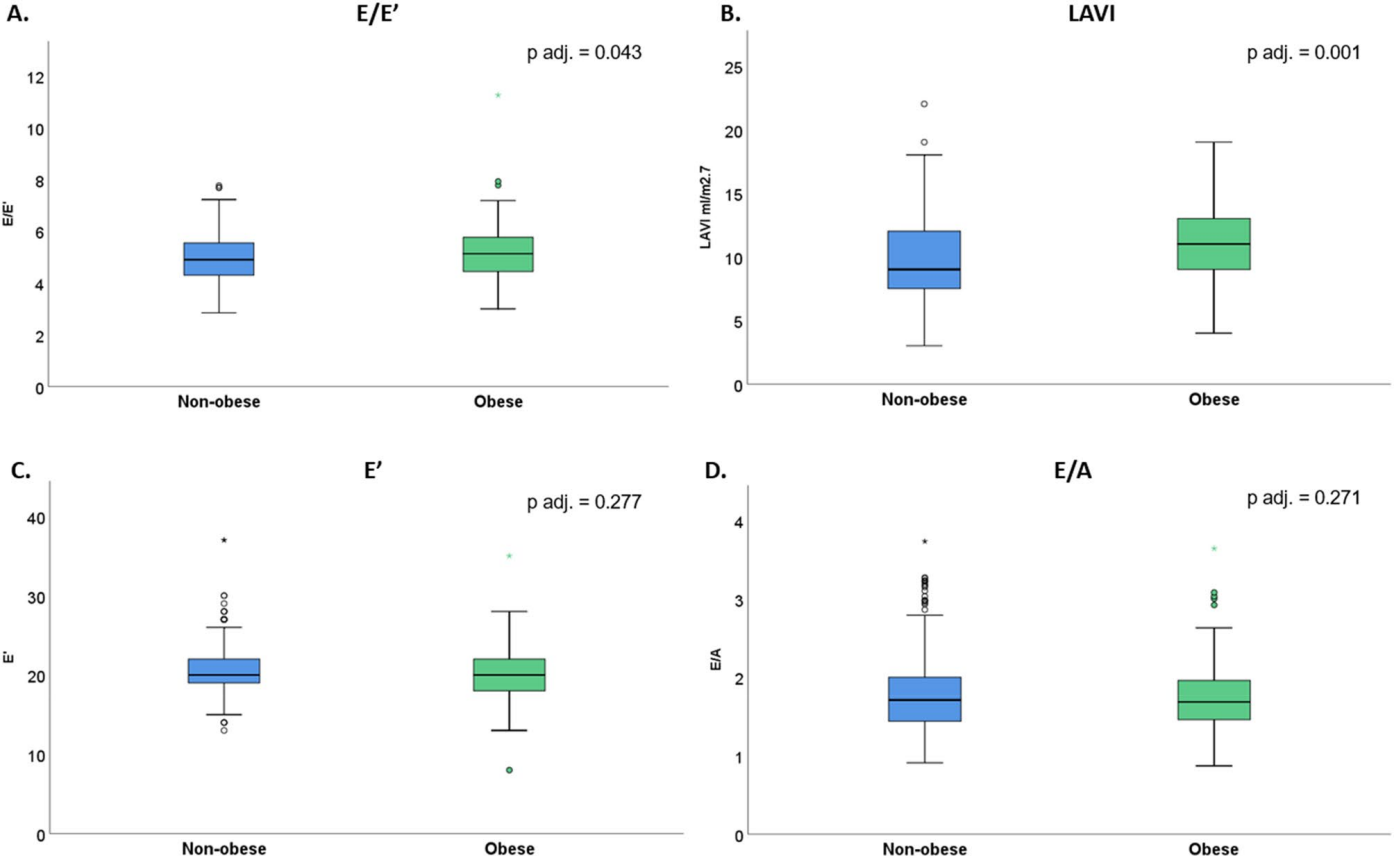
Parameters of diastolic function in obese and non-obese children. (**A**) E/E′ indicates the ratio between mitral peak velocity of early filling and peak early diastolic tissue velocities of mitral annulus. (**B**) Left atrial volume indexed to height. (**C**) E′ indicates peak early diastolic tissue velocities of mitral annulus. (**D**) E/A indicates the ratio between mitral peak velocity of early diastolic filling and atrial filling.

Systolic left and right systolic ventricular function were comparable between obese and non-obese children. In turn, measures of diastolic function differed significantly. Obese children had a significantly higher E/E′ ratio (5.18 vs. 4.93, p_adjusted_ = 0.043) and larger left atrial volume index (LAVI) (11.0 vs 9.6 ml/m^2.7^; p_adjusted_ = 0.001) than non-obese children; E′ and E/A ratio did not differ between the two groups (Table 2, Fig. 4). The findings of a propensity score matched analysis based on age and sex were consistent with the adjusted analysis (Supplementary Table 1). The effect of obesity on LVH was consistent with no significant interaction according to sex. Similarly, there was no significant interaction in the E/E′ ratio. In contrast, the correlation between obesity and left atrial enlargement was particularly pronounced in girls as compared to boys (Supplementary Table 2).

Echocardiographic data stratified by presence or absence of obesity and LVH is shown in Supplementary Table 3. LV filling pressures as expressed by the E/E′ ratio were increased in obese children irrespective of presence or absence of LVH, whereas LAVI increased across a gradient from non-obese children with no LVH to non-obese children with LVH or obese children with no LVH, to obese children with LVH.

Measures of left ventricular geometry and function according to age categories are provided separately for girls and boys in Supplemental Tables 4 and 5, respectively.

## Discussion

In a representative sample of randomly selected children in the range of 5–16 years of age from a cross-sectional study, we found an association between obesity and both LVH and echocardiographic determinants of diastolic dysfunction. Obese children had greater LVM, greater E/E′ ratio, and a greater left atrial volume than non-obese children.The E/E′ ratio has been shown to correlate with the mean pulmonary capillary wedge pressure and the LV filling pressures^17^. In our study, LV filling pressures were increased in children with obesity irrespective of evidence of LVH, whereas LAVI increased across a gradient with highest values in obese children with LVH (Supplementary Table 3).

We found a linear correlation of BMI z-score and LVMI and almost fourfold increased prevalence of LVH in obese as compared to non-obese children. Consistent with the results of a small prospective study including 101 children with a mean age of 13.5 years, obese children were found to have a greater septal and posterior wall thickness^18^. The impact of obesity on LV geometry may take effect at an early stage in life. Data from the Generation R Study including 974 children with longitudinal echocardiographic follow-up indicated, that overweight and obese children develop cardiac adaptions similar to those of obese adults as young as 2 years of age^19^. In addition, data from the Framingham Cohort Study showed that the number of years lived with obesity is directly associated with the risk of mortality^20^.

Echocardiographic indicators of LV diastolic function were significantly different between obese and non-obese children in our study. In particular, we found greater LV diastolic filling pressures (E/E′) and larger left atrial volume in obese as compared to non-obese children. The latter finding corroborated the results of previous reports^5,18^. In contrast to the study by Mangner and colleagues, we did not find differences in mitral inflow early-to-late diastolic flow (E/A) ratio in obese versus non-obese children^18^. Evidence of the effect of LVH on LV diastolic dysfunction is controversial. In one study including 74 severely obese patients with a mean age of 37 ± 8 years, increased LVMI correlated with elevated LV filling pressures^21^. However, recent evidence suggested an increased E/E′ ratio as an early marker of obesity cardiomyopathy in obese children even in the absence of LVH. In one small cross-sectional study including 32 obese and 30 non-obese children with a mean age of 10.6 years, obese children were found to have increased LV filling pressures as expressed by the E/E′ ratio (9.0 ± 1.6 versus 6.9 ± 1.4; p value = 0.001) compared to non-obese subjects even after exclusion of cases with LVH^22^. Our findings corroborate this observation with numerically higher LV filling pressures in obese as compared to non-obese children irrespective of echocardiographic evidence of LVH.

The impact of obesity on LVH and diastolic dysfunction in adults is explained by hemodynamic and endocrinologic alterations related to obesity^23^. Excessive fat tissue accumulation promotes the growth of peripheral blood vessels eventually resulting in an increase in cardiac output. Hemodynamic overload leads to increase in diastolic filling pressures, LV wall stress and LVH^24^. Pericardial and intracellular fat increases heart muscle, stiffens the LV, and compromises diastolic function^25^. Various neurohormonal and metabolic factors related to obesity further contribute to cardiac remodeling^26^. However, the importance of these pathophysiological mechanisms in children is incompletely understood.

Almost all of these unfavorable alterations of LV geometry and function are responsive to weight loss^24,27,28^. The most robust data in this context comes from clinical outcomes after bariatric surgery. Ippisch and colleagues reported data from 38 adolescents with a mean age of 16 years evaluated before and after bariatric surgery. After a mean follow up of 10 months and a substantial weight loss of 59 ± 15 kg, echocardiographic follow up showed a reduction of LVMI (54 ± 13 g/m^2.7^ to 42 ± 10 g/m^2.7^, p < 0.0001), a reduction in the prevalence of LVH from 28 to 3%, and improvement in diastolic function^29^. Moreover, prospective data of 62 children with a mean duration of follow up of 3.2 years showed normalization of global longitudinal strain after weight reduction^28^.

The potential reversibility of LV dysfunction associated with obesity highlights the importance of early diagnosis and treatment of obesity in children. Echocardiography is currently not routinely recommended in the management of obese children, and is reserved for children with arterial hypertension^30–33^. Implementation of evidence based interventions and early educational programs focused on healthy life style promotion in children may represent a window of opportunity to prevent HFpEF in adulthood.

The findings of the present analysis need to be interpreted in light of several limitations. First, there is no standard definition of diastolic dysfunction in children and adolescents. While our study indicates statistically significant differences in parameters of diastolic function, the clinical relevance of this difference remains unclear. Second, one third of children included into the cross-sectional study were excluded from the present analysis due to missing echocardiographic data; this may have introduced a selection bias of the studied cohort. However, an analysis using multiple imputation showed consistent results with the main findings. Third, E′ was measured at the lateral mitral annulus only, which may have led to an underestimation of LV filling pressures. Fourth, blood pressure was not prospectively recorded and may have contributed to the development of LVH and diastolic dysfunction. However, there is evidence suggesting that LVH and diastolic dysfunction in obese children are present even before the development of arterial hypertension^35^. Fifth, reproducibility testing is not available; however, all analyses were performed in agreement with a pre-defined analysis plan following the left chamber quantification guidelines and with the supervision of one cardiologist (ES), reducing the risk of uncontrolled variability. Sixth, children were not screened for disorders of the endocrine system or chromosomal disorders, which may have led to confounding of our data. And finally, our study represents a cross-sectional survey. Longitudinal data is needed to delineate the risk of transition from subclinical diastolic dysfunction in childhood to HFpEF later on in life.

In conclusion, in a cross-sectional study among children, obesity was associated with increased LVM, LVH, increased LV filling pressures, and increased left atrial volume. Early signs of compromised diastolic function in obese children highlight the importance of healthy life style promotion in children.

## Supplementary Information

Supplementary Information.

## Abbreviations

BMI: Body mass index
EF: Ejection fraction
E/A: Mitral inflow early-to-late diastolic flow ratio
E′: Peak early diastolic tissue velocities of mitral annulus
LV: Left ventricle
LVH: Left ventricular hypertrophy
LVM: Left ventricular mass
LVMI: Left ventricular mass index
LV S′: Tissue Doppler-derived peak systolic velocity of LV lateral wall
HFpEF: Heart failure with preserved ejection fraction
RV S′: Tissue Doppler-derived peak systolic velocity at RV free wall
TAPSE: Tricuspid annular plane systolic excursion
WHO: World Health Organization
