# Supplementary material for: The impact of obesity on left ventricular hypertrophy and diastolic dysfunction in children and adolescents
Source: Sci Rep. 2021 Jun 22;11:13022. doi: 10.1038/s41598-021-92463-x (PMC8219764; doi:10.1038/s41598-021-92463-x)
Supplement: Supplementary file 1 — Supplementary Information. [file 41598_2021_92463_MOESM1_ESM.docx]

**The Impact of Obesity on Left Ventricular Hypertrophy and Diastolic Dysfunction in Children and Adolescents**

**Supplementary Appendix**

ClinicalTrials.gov Identifier: NCT02353663

Joanna Bartkowiak, MD^1*^; Ernest Spitzer, MD^2,3*^; Reto Kurmann, MD^4^; Fabian Zürcher, MD^1^; Peter Krähenmann, MD^5^; Victoria Garcia-Ruiz, MD^2^; Jorge Mercado, MD^6^; Christoph Ryffel, MD^1^; Sylvain Losdat, PhD^7^; Nassip Llerena, MD^8^; Pedro Torres, MD^6,8^; Jonas Lanz, MD^1^; Martin Stocker, MD, MME^4^; Ben Ren, MD, PhD^2,3^; Martin Glöckler, MD^9^; Thomas Pilgrim, MD, MSc^1^

*the first two authors contributed equally to this manuscript

^1^Department of Cardiology, Inselspital, University of Bern, Bern, Switzerland; ^2^Cardialysis, Rotterdam, The Netherlands; ^3^ Thoraxcenter, Erasmus University Medical Center, Rotterdam, The Netherlands, ^4^Department of Cardiology, Cantonal Hospital Lucerne, Switzerland; ^5^Department of Cardiology, Cantonal Hospital St. Gallen, Switzerland; ^6^Institute of Cardiology CardioSalud, Arequipa, Peru; ^7^CTU Bern, University of Bern, Switzerland; ^8^ National Hospital Carlos Alberto Seguín Escobedo, Arequipa, Peru; ^9^Department of Pediatric Cardiology, Inselspital, University of Bern, Bern, Switzerland

Corresponding author: Thomas Pilgrim, MD, MSc

Department of Cardiology

Inselspital

Bern University Hospital

University of Bern

CH-3010 Bern

Phone: 0041 31 632 08 27

Fax: 0041 31 632 47 70

Mail: [thomas.pilgrim@insel.ch](mailto:thomas.pilgrim@insel.ch)

**Abbreviations**

BMIz - Z-score for body mass index

LV – Left ventricle

LVM - Left ventricular mass

LVMI - Left ventricular mass index

WHO - World Health Organization

1. Computation of the left ventricular mass (LVM) and left ventricular mass index (LVMI)

LVM was computed using following formula:

LVM= 0.81*(1.04*(intraventricular septal wall thickness + posterior wall thickness + LV end diastolic internal dimension)^3^ – (LV end diastolic internal dimension)^3^ )+ 0.6 g ^1^

LVM was then indexed to the 2.7 power of height in meter, as recommended by the National High Blood Pressure Education Program Working Group on High Blood Pressure in Children and Adolescents ^2^

LVMI = $\frac{LVM}{{height}^{2.7}}$ g/m^2.7^

1. Computation of the z-score for body mass index (BMIz), as recommended by World Health Organization (WHO) ^3^ ^4^.

Z-score for BMI was calculated using following formula:

$BMIz= \frac{{[\frac{y}{M\left( t \right)}]}^{L(t)}-1}{S\left( t \right)L(t)}$ if │BMIz│ ≤ 3

Where weight is denoted by *y*. The tabulated fitted values of Box-Cox power, median and coefficient of variation corresponding to age *t* are denoted by *L(t)*, *M(t)* and *S(t)*, respectively.

BMIz at the extreme tails (beyond ±3 SD) was adjusted using following formula:

$3+(\frac{y-SD3pos}{SD23pos})$ if BMIz > 3

$\left( -3 \right)+(\frac{y-SD3neg}{SD23neg})$ if BMIz < (-3)

Where

- ${SD3pos = M(t)x(1+ L\left( t \right)\mathrm{xS}\left( t \right)x\left( 3 \right))}^{1/L(t)}$
- ${SD3neg = M(t)x(1+ L\left( t \right)\mathrm{xS}\left( t \right)x\left( -3 \right))}^{1/L(t)}$
- ${SD23pos = M(t)x(1+ L\left( t \right)\mathrm{xS}\left( t \right)x\left( 3 \right))}^{1/L(t)}- {M(t)x(1+ L\left( t \right)\mathrm{xS}\left( t \right)x\left( 2 \right))}^{1/L(t)}$
- ${SD23neg = M(t)x(1+ L\left( t \right)\mathrm{xS}\left( t \right)x\left( -2 \right))}^{1/L(t)}- {M(t)x(1+ L\left( t \right)\mathrm{xS}\left( t \right)x\left( -3 \right))}^{1/L(t)}$

**Supplementary Table 1. Propensity score matched analysis based age and sex using ≥ 5 neighbours for left ventricular hypertrophy and diastolic parameters.**

|  | **All children**  (n = 681) | **Non-obese (BMIz ≤ 2)**  (n = 531) | **Obese (BMIz > 2)**  (n = 150) | **P-value**  **(Nearest-neighbour PS matching with ≥5 neighbours)** |
| --- | --- | --- | --- | --- |
| LV Hypertrophy, n (%) | 40 (5.87%) | 19 (3.58%) | 21 (14.0%) | <0·001 |
| E/E’ ratio, mean (SD) | 4.99 (0.898) | 4.93 (0.845) | 5.18 (1.050) | 0.077 |
| LAVI (ml/m^2.7^), mean (SD) | 9.9 (3.030) | 9.6 (2.910) | 11.0 (3.170) | 0.007 |
| E’ (cm/s), mean (SD) | 20.4 (3.080) | 20.5 (2.960) | 20.2 (3.460) | 0.420 |
| E/A ratio, mean (SD) | 1.77 (0.453) | 1.78 (0.453) | 1.77 (0.455) | 0.994 |
| BMIz = BMI z-score, E= inflow early diastolic flow velocity, E’ = mitral lateral peak early diastolic tissue velocities, E/A - inflow early-to-late diastolic flow ratio, LAVI = left atrial volume index | | | | |

**Supplementary Table 2.** E**ffect of obesity on LVH and diastolic dysfunction in females and males.**

|  | Boys  (n = 341) | Non-obese (BMIz ≤ 2) (n = 234) | Obese (BMIz > 2) (n = 107) | **P-value (uncorrected)** | Girls  (n = 340) | Non-obese (BMIz ≤ 2) (n = 297) | Obese (BMIz > 2) (n = 43) | **P-value (uncorrected)** | **P-value (interaction)** |
| --- | --- | --- | --- | --- | --- | --- | --- | --- | --- |
| LV-hypertrophy, n (%) | 20 (5.87%) | 6 (2.56%) | 14 (13.1%) | 0.001 | 20 (5.88%) | 13 (4.38%) | 7 (16.3%) | 0.004 | 0.675 |
| Diastolic LV function |  |  |  |  |  |  |  |  |  |
| E/E’ ratio, mean (SD) | 5.07 (0.931) | 5.01 (0.833) | 5.19 (1.11) | 0.084 | 4.91 (0.858) | 4.87 (0.850) | 5.14 (0.885) | 0.066 | 0.623 |
| LAVI (ml/m^2.7^), mean (SD) | 10.1 (3.09) | 9.8 (3.07) | 10.6 (3.05) | 0.018 | 9.7 (2.96) | 9.37 (2.76) | 11.8 (3.34) | <0.001 | 0.008 |
| E’ (cm/s), mean (SD) | 20.5 (3.04) | 20.7 (2.86) | 20.0 (3.37) | 0.047 | 20.4 (3.12) | 20.3 (3.03) | 20.8 (3.67) | 0.321 | 0.050 |
| E/A ratio, mean (SD) | 1.81 (0.463) | 1.85 (0.474) | 1.74 (0.430) | 0.051 | 1.73 (0.440) | 1.72 (0.428) | 1.83 (0.511) | 0.138 | 0.019 |
| BMI z-score, E= inflow early diastolic flow velocity, E’ = mitral lateral peak early diastolic tissue velocities, E/A - inflow early-to-late diastolic flow ratio, LAVI = left atrial volume index, LV = left ventricle, SD = standard deviation | | | | | | | | | |

**Supplementary Table 3.** **Echocardiographic findings stratified by presence or absence of obesity and left ventricular hypertrophy**

|  | LVH hypertrophy (n = 40) | Non-obese (BMIz ≤ 2) (n= 19) | Obese  (BMIz > 2)  (n = 21) | P-value (uncorrected) | No LV hypertrophy  (n = 641) | Non-obese (BMIz ≤ 2) (n = 512) | Obese  (BMIz > 2)  (n = 129) | P-value (uncorrected) | P-value (interaction) |
| --- | --- | --- | --- | --- | --- | --- | --- | --- | --- |
|  | mean (sd) | mean (sd) | mean (sd) |  | mean (sd) | mean (sd) | mean (sd) |  |  |
| **CARDIAC GEOMETRY** |  |  |  |  |  |  |  |  |  |
| LVEDD (cm) | 4.10 (0.475) | 4.15 (0.529) | 4.05 (0.428) | 0.515 | 3.90 (0.478) | 3.90 (0.489) | 3.92 (0.430) | 0.643 | 0.447 |
| LVESD (cm) | 2.47 (0.323) | 2.50 (0.342) | 2.45 (0.311) | 0.607 | 2.39 (0.323) | 2.39 (0.329) | 2.37 (0.300) | 0.461 | 0.786 |
| SWTd (cm) | 0.895 (0.170) | 0.929 (0.209) | 0.864 (0.122) | 0.174 | 0.721 (0.150) | 0.710 (0.150) | 0.764 (0.143) | 0.000 | 0.018 |
| PWTd (cm) | 0.785 (0.133) | 0.790 (0.127) | 0.781 (0.142) | 0.808 | 0.640 (0.117) | 0.632 (0.120) | 0.670 (0.104) | 0.001 | 0.226 |
| RWT | 0.390 (0.090) | 0.390 (0.102) | 0.389 (0.080) | 0.989 | 0.333 (0.076) | 0.330 (0.078) | 0.346 (0.065) | 0.031 | 0.513 |
| LVMI (g/m^2.7^) | 48.2 (8.05) | 45.8 (5.03) | 50.4 (9.7) | 0.020 | 29.3 (6.48) | 28.1 (6.14) | 33.8 (5.79) | <0.001 | 0.575 |
| Right atrial area ES (cm^2^) | 10.4 (2.62) | 10.7 (2.70) | 10.0 (2.54) | 0.339 | 10.2 (2.30) | 10.1 (2.27) | 10.7 (2.34) | 0.008 | 0.090 |
| Basal RV diameter 4C (cm) | 3.03 (0.627) | 3.04 (0.671) | 3.02 (0.596) | 0.917 | 3.07 (0.597) | 3.05 (0.606) | 3.15 (0.558) | 0.103 | 0.558 |
| Base-to-apex length 4C (cm) | 5.97 (1.24) | 5.99 (1.50) | 5.95 (1.000) | 0.901 | 6.24 (0.924) | 6.19 (0.909) | 6.43 (0.958) | 0.015 | 0.397 |
| Mid RV diameter 4C (cm) | 2.76 (0.720) | 3.02 (0.779) | 2.51 (0.581) | 0.018 | 2.78 (0.575) | 2.75 (0.577) | 2.93 (0.547) | 0.019 | 0.003 |
| **CARDIAC FUNCTION** |  |  |  |  |  |  |  |  |  |
| **Systolic LV Function** |  |  |  |  |  |  |  |  |  |
| LV S’ (cm/s) | 11.5 (1.65) | 11.5 (1.43) | 11.5 (1.86) | 0.997 | 12.2 (1.89) | 12.2 (1.89) | 12.0 (1.87) | 0.362 | 0.783 |
| EF (%) | 69.4 (6.11) | 70.3 (5.67) | 68.7 (6.54) | 0.437 | 69.4 (6.31) | 69.2 (6.31) | 70.2 (6.27) | 0.109 | 0.223 |
| **Diastolic LV function** |  |  |  |  |  |  |  |  |  |
| E/E’ ratio | 5.11 (0.827) | 4.96 (0.819) | 5.25 (0.832) | 0.321 | 4.98 (0.902) | 4.93 (0.846) | 5.17 (1.08) | 0.008 | 0.872 |
| LAVI (ml/m^2.7^) | 11.7 (3.48) | 11.0 (2.95) | 12.4 (3.86) | 0.144 | 9.8 (2.96) | 9.5 (2.90) | 10.8 (3.02) | 0.000 | 0.865 |
| E’ (cm/s) | 20.0 (2.78) | 19.8 (2.27) | 20.0 (3.23) | 0.833 | 20.4 (3.09) | 20.5 (2.98) | 20.2 (3.51) | 0.382 | 0.645 |
| E/A ratio | 1.78 (0.501) | 1.78 (0.491) | 1.78 (0.521) | 0.990 | 1.77 (0.451) | 1.78 (0.452) | 1.77 (0.445) | 0.825 | 0.938 |
| **RV Function** |  |  |  |  |  |  |  |  |  |
| RV S’ (cm/s) | 14.1 (1.93) | 14.4 (2.09) | 13.7 (1.76) | 0.270 | 14.3 (1.82) | 14.3 (1.85) | 14.1 (1.70) | 0.278 | 0.463 |
| TAPSE (cm) | 2.02 (0.312) | 2.07 (0.328) | 1.98 (0.300) | 0.353 | 2.13 (0.297) | 2.14 (0.300) | 2.11 (0.282) | 0.292 | 0.566 |
| 4C = four-chamber view, BMIz = BMI z-score, E= inflow early diastolic flow velocity, E’ = mitral lateral peak early diastolic tissue velocities, EF = ejection fraction, ES = end systolic, E/A - inflow early-to-late diastolic flow ratio, LAVI = left atrial volume index, LV = left ventricle, LVEDD = left ventricular end-diastolic diameter, LVESD = left ventricular end-systolic diameter, LVMI – left ventricular mass index, LV S’ = tissue Doppler-derived peak systolic velocity of LV wall, PWTd = posterior wall thickness (end diastolic), RV = right ventricle, RV S’ = tissue Doppler-derived peak systolic velocity at RV free wall, RWT = relative wall thickness, SWTd = septal wall thickness (end diastolic), sd = standard deviation, TAPSE = tricuspid annular plane systolic excursion | | | | | | | | | |

**Supplementary Table 4. Left ventricular geometry and function according to age categories in girls**.

|  | All girls  (n = 340) | Non-obese ≤10y  (n = 50) | Obese  ≤10y  (n = 23) | **P-value** | Non-obese >10 & ≤14y (n = 131) | Obese  >10 & ≤14y  (n = 15) | **P-value** | Non-obese >14y  (n = 116) | Obese  ≥14y  (n = 5) | **P-value** |
| --- | --- | --- | --- | --- | --- | --- | --- | --- | --- | --- |
|  | mean (sd)  or n (%) | mean (sd)  or n (%) | mean (sd)  or n (%) |  | mean (sd)  or n (%) | mean (sd)  or n (%) |  | mean (sd)  or n (%) | mean (sd)  or n (%) |  |
| LVH (n) | 20 (5.88%) | 3 (6.00%) | 3 (13.0%) | 0.320 | 9 (6.87%) | 3 (20.0%) | 0.095 | 1 (0.862%) | 1 (20.0%) | 0.025 |
| LVEDD (cm) | 3.92 (0.480) | 3.38 (0.283) | 3.55 (0.353) | 0.077 | 3.96 (0.423) | 4.24 (0.381) | 0.009 | 4.11 (0.422) | 4.47 (0.222) | 0.048 |
| LVESD (cm) | 2.40 (0.314) | 2.14 (0.287) | 2.19 (0.249) | 0.472 | 2.38 (0.280) | 2.53 (0.251) | 0.046 | 2.53 (0.284) | 2.72 (0.260) | 0.141 |
| SWTd (cm) | 0.711 (0.155) | 0.646 (0.145) | 0.687 (0.128) | 0.295 | 0.725 (0.162) | 0.760 (0.154) | 0.405 | 0.719 (0.152) | 0.790 (0.104) | 0.314 |
| PWTd (cm) | 0.631 (0.119) | 0.580 (0.106) | 0.605 (0.099) | 0.399 | 0.635 (0.130) | 0.677 (0.138) | 0.196 | 0.647 (0.107) | 0.682 (0.117) | 0.512 |
| RWT | 0.328 (0.080) | 0.344 (0.060) | 0.345 (0.071) | 0.987 | 0.327 (0.087) | 0.322 (0.075) | 0.828 | 0.321 (0.081) | 0.305 (0.051) | 0.670 |
| LVMI (g/m^2.7) | 28.4 (7.47) | 29.8 (6.97) | 35.3 (8.32) | 0.002 | 27.7 (7.71) | 34.2 (9.21) | 0.001 | 26.3 (5.58) | 32.2 (7.06) | 0.070 |
| Right atrial area ES (cm2) | 10.0 (2.08) | 8.17 (1.30) | 8.68 (1.14) | 0.274 | 10.2 (1.97) | 11.3 (2.44) | 0.041 | 10.8 (1.93) | 12.0 (1.98) | 0.202 |
| Basal RV diameter 4C (cm) | 3.02 (0.633) | 2.70 (0.405) | 2.86 (0.483) | 0.307 | 3.04 (0.667) | 3.31 (0.760) | 0.128 | 3.15 (0.633) | 3.11 (0.844) | 0.897 |
| Base-to-apex lenght 4C (cm) | 6.19 (0.853) | 5.34 (0.692) | 5.73 (0.788) | 0.052 | 6.34 (0.761) | 6.72 (0.962) | 0.088 | 6.46 (0.725) | 5.73 (0.825) | 0.060 |
| Mid RV diameter 4C (cm) | 2.75 (0.553) | 2.40 (0.397) | 2.64 (0.515) | 0.180 | 2.86 (0.519) | 2.93 (0.815) | 0.706 | 2.79 (0.557) | 3.44 (0.530) | 0.085 |
| LV S’ (cm/s) | 12.2 (1.88) | 11.5 (2.22) | 11.5 (1.31) | 0.929 | 12.2 (1.87) | 12.1 (1.75) | 0.817 | 12.5 (1.72) | 13.2 (2.49) | 0.437 |
| EF (%) | 69.5 (6.08) | 67.3 (7.16) | 69.4 (5.02) | 0.176 | 70.5 (5.81) | 70.8 (5.52) | 0.868 | 69.1 (6.01) | 69.6 (4.88) | 0.847 |
| E/E’ ratio | 4.91 (0.858) | 4.91 (0.838) | 5.09 (0.965) | 0.394 | 4.90 (0.869) | 5.18 (0.869) | 0.234 | 4.82 (0.839) | 5.24 (0.652) | 0.290 |
| LAVI (ml/m^2.7^) | 9.7 (2.96) | 10.1 (3.33) | 13.3 (2.94) | 0.000 | 8.95 (2.45) | 10.3 (3.12) | 0.091 | 9.5 (2.73) | 8.63 (0.919) | 0.521 |
| E’ (cm/s) | 20.4 (3.12) | 21.1 (4.24) | 21.4 (3.93) | 0.710 | 20.2 (2.76) | 20.0 (3.55) | 0.780 | 20.1 (2.66) | 20.6 (2.79) | 0.709 |
| E/A ratio | 1.73 (0.440) | 1.86 (0.425) | 1.91 (0.571) | 0.651 | 1.69 (0.457) | 1.62 (0.249) | 0.566 | 1.69 (0.386) | 2.08 (0.685) | 0.050 |
| RV S’ (cm/s) | 14.4 (1.87) | 14.1 (1.77) | 13.5 (1.57) | 0.199 | 14.1 (1.81) | 14.3 (2.13) | 0.789 | 15.0 (1.85) | 15.0 (2.65) | 0.971 |
| TAPSE (cm) | 2.15 (0.291) | 1.98 (0.236) | 1.93 (0.230) | 0.444 | 2.15 (0.283) | 2.23 (0.314) | 0.322 | 2.26 (0.272) | 2.22 (0.307) | 0.695 |
| Adj. p value = p value adjusted for age and sex, 4C = four-chamber view, BMIz = BMI z-score, E= inflow early diastolic flow velocity, E’ = mitral lateral peak early diastolic tissue velocities, EF = ejection fraction, ES = end systolic, E/A - inflow early-to-late diastolic flow ratio, LAVI = left atrial volume index, LV = left ventricle, LVEDD = left ventricular end-diastolic diameter, LVESD = left ventricular end-systolic diameter, LVMI – left ventricular mass index, LV S’ = tissue Doppler-derived peak systolic velocity of LV wall, PWTd = posterior wall thickness (end diastolic), RV = right ventricle, RV S’ = tissue Doppler-derived peak systolic velocity at RV free wall, RWT = relative wall thickness, SWTd = septal wall thickness (end diastolic), sd = standard deviation, TAPSE = tricuspid annular plane systolic excursion. | | | | | | | | | | |

**Supplementary Table 5. Left ventricular geometry and function according to age categories in boys.**

|  | All boys  (n = 341) | Non-obese ≤10y  (n = 79) | Obese  ≤10y  (n = 49) | **P-value** | Non-obese >10 & ≤14y (n = 90) | Obese  >10 & ≤14y  (n = 50) | **P-value** | Non-obese >14y  (n = 65) | Obese  >14y  (n = 8) | **P-value** |
| --- | --- | --- | --- | --- | --- | --- | --- | --- | --- | --- |
|  | mean (sd)  or n (%) | mean (sd)  or n (%) | mean (sd)  or n (%) |  | mean (sd)  or n (%) | mean (sd)  or n (%) |  | mean (sd)  or n (%) | mean (sd)  or n (%) |  |
| LVH (n) | 20 (5.87%) | 3 (3.80%) | 10 (20.4%) | 0.006 | 1 (1.11%) | 3 (6.00%) | 0.137 | 2 (3.08%) | 1 (12.5%) | 0.243 |
| LVEDD (cm) | 3.91 (0.480) | 3.53 (0.440) | 3.79 (0.370) | 0.000 | 3.87 (0.381) | 4.02 (0.335) | 0.026 | 4.35 (0.383) | 4.49 (0.294) | 0.333 |
| LVESD (cm) | 2.39 (0.333) | 2.23 (0.308) | 2.37 (0.273) | 0.012 | 2.34 (0.284) | 2.37 (0.296) | 0.658 | 2.66 (0.328) | 2.59 (0.383) | 0.537 |
| SWTd (cm) | 0.751 (0.156) | 0.629 (0.112) | 0.743 (0.129) | 0.000 | 0.738 (0.136) | 0.852 (0.135) | 0.000 | 0.837 (0.159) | 0.816 (0.122) | 0.679 |
| PWTd (cm) | 0.665 (0.125) | 0.592 (0.107) | 0.673 (0.094) | 0.000 | 0.644 (0.109) | 0.733 (0.117) | 0.000 | 0.719 (0.140) | 0.719 (0.114) | 0.994 |
| RWT | 0.345 (0.076) | 0.344 (0.097) | 0.360 (0.070) | 0.258 | 0.335 (0.062) | 0.367 (0.063) | 0.016 | 0.334 (0.075) | 0.323 (0.064) | 0.702 |
| LVMI (g/m^2.7) | 32.3 (7.94) | 31.7 (6.50) | 37.6 (6.74) | 0.000 | 29.5 (6.30) | 35.9 (8.49) | 0.000 | 29.6 (7.16) | 37.5 (17.8) | 0.004 |
| Right atrial area ES (cm2) | 10.4 (2.51) | 8.23 (1.53) | 9.7 (2.06) | 0.000 | 10.3 (1.84) | 11.5 (1.85) | 0.001 | 12.4 (2.44) | 14.3 (2.49) | 0.010 |
| Basal RV diameter 4C (cm) | 3.12 (0.560) | 2.82 (0.440) | 3.02 (0.467) | 0.035 | 3.05 (0.472) | 3.24 (0.478) | 0.040 | 3.50 (0.623) | 3.65 (0.735) | 0.430 |
| Base-to-apex lenght 4C (cm) | 6.25 (1.03) | 5.53 (0.837) | 6.05 (0.965) | 0.003 | 6.16 (0.911) | 6.78 (0.794) | 0.000 | 6.94 (0.927) | 7.04 (0.982) | 0.777 |
| Mid RV diameter 4C (cm) | 2.81 (0.616) | 2.40 (0.532) | 2.71 (0.480) | 0.012 | 2.76 (0.462) | 2.94 (0.458) | 0.155 | 3.33 (0.592) | 3.24 (0.883) | 0.725 |
| LV S’ (cm/s) | 12.1 (1.88) | 11.1 (1.48) | 11.4 (1.57) | 0.368 | 12.5 (1.86) | 12.5 (2.21) | 0.943 | 12.9 (1.72) | 12.4 (1.60) | 0.434 |
| EF (%) | 69.3 (6.50) | 67.2 (6.31) | 67.9 (6.16) | 0.570 | 70.5 (5.85) | 71.8 (6.19) | 0.244 | 69.0 (6.66) | 72.0 (10.1) | 0.214 |
| E/E’ ratio | 5.07 (0.931) | 5.11 (0.787) | 5.20 (1.34) | 0.632 | 4.91 (0.821) | 5.23 (0.897) | 0.055 | 5.02 (0.898) | 4.92 (0.761) | 0.783 |
| LAVI (ml/m^2.7^) | 10.1 (3.09) | 11.6 (3.05) | 11.3 (2.92) | 0.554 | 9.07 (2.82) | 10.1 (3.03) | 0.047 | 8.46 (2.29) | 9.9 (3.88) | 0.259 |
| E’ (cm/s) | 20.5 (3.04) | 20.4 (2.79) | 20.0 (4.15) | 0.433 | 21.0 (2.81) | 19.9 (2.44) | 0.042 | 20.6 (2.99) | 20.2 (3.41) | 0.789 |
| E/A ratio | 1.81 (0.463) | 1.93 (0.488) | 1.68 (0.404) | 0.003 | 1.81 (0.467) | 1.80 (0.450) | 0.892 | 1.79 (0.460) | 1.81 (0.459) | 0.943 |
| RV S’ (cm/s) | 14.1 (1.79) | 13.7 (1.93) | 13.9 (1.49) | 0.545 | 14.0 (1.69) | 14.3 (1.74) | 0.515 | 14.8 (1.80) | 14.6 (1.81) | 0.717 |
| TAPSE (cm) | 2.09 (0.303) | 1.97 (0.276) | 2.02 (0.267) | 0.358 | 2.08 (0.298) | 2.13 (0.278) | 0.291 | 2.27 (0.303) | 2.34 (0.246) | 0.554 |
| Adj. p value = p value adjusted for age and sex, 4C = four-chamber view, BMIz = BMI z-score, E= inflow early diastolic flow velocity, E’ = mitral lateral peak early diastolic tissue velocities, EF = ejection fraction, ES = end systolic, E/A - inflow early-to-late diastolic flow ratio, LAVI = left atrial volume index, LV = left ventricle, LVEDD = left ventricular end-diastolic diameter, LVESD = left ventricular end-systolic diameter, LVMI – left ventricular mass index, LV S’ = tissue Doppler-derived peak systolic velocity of LV wall, PWTd = posterior wall thickness (end diastolic), RV = right ventricle, RV S’ = tissue Doppler-derived peak systolic velocity at RV free wall, RWT = relative wall thickness, SWTd = septal wall thickness (end diastolic), sd = standard deviation, TAPSE = tricuspid annular plane systolic excursion. | | | | | | | | | | |

**REFERENCES:**

1. Brady TM. The Role of Obesity in the Development of Left Ventricular Hypertrophy Among Children and Adolescents. *Curr Hypertens Rep*. 2016;18:1–7.

2. Roccella EJ. Update on the 1987 task force report on high blood pressure in children and adolescents: A working group report from the national high blood pressure education program. *Pediatrics*. 1996;98:649–658.

3. WHO Child Growth Standards. *Dev Med Child Neurol*. 2009;51:1002–1002.

4. WHO bmi charts [Internet]. [cited 2020 Dec 1];Available from: https://www.who.int/toolkits/child-growth-standards/standards/body-mass-index-for-age-bmi-for-age
